# Drug–target interaction prediction based on protein features, using wrapper feature selection

**DOI:** 10.1038/s41598-023-30026-y

**Published:** 2023-03-03

**Authors:** Hengame Abbasi Mesrabadi, Karim Faez, Jamshid Pirgazi

**Affiliations:** 1 Faculty of Computer and Information Technology Engineering, Qazvin Branch, Islamic Azad University, Qazvin, Iran; 2 Department of Electrical Engineering, Amirkabir University of Technology (Tehran Polytechnic), Tehran, Iran; 3 Department of Computer Engineering, University of Science and Technology of Mazandaran, Behshahr, Iran

**Keywords:** Drug discovery, Diseases, Medical research, Engineering

## Abstract

Drug–target interaction prediction is a vital stage in drug development, involving lots of methods. Experimental methods that identify these relationships on the basis of clinical remedies are time-taking, costly, laborious, and complex introducing a lot of challenges. One group of new methods is called computational methods. The development of new computational methods which are more accurate can be preferable to experimental methods, in terms of total cost and time. In this paper, a new computational model to predict drug–target interaction (DTI), consisting of three phases, including feature extraction, feature selection, and classification is proposed. In feature extraction phase, different features such as EAAC, PSSM and etc. would be extracted from sequence of proteins and fingerprint features from drugs. These extracted features would then be combined. In the next step, one of the wrapper feature selection methods named IWSSR, due to the large amount of extracted data, is applied. The selected features are then given to rotation forest classification, to have a more efficient prediction. Actually, the innovation of our work is that we extract different features; and then select features by the use of IWSSR. The accuracy of the rotation forest classifier based on tenfold on the golden standard datasets (enzyme, ion channels, G-protein-coupled receptors, nuclear receptors) is as follows: 98.12, 98.07, 96.82, and 95.64. The results of experiments indicate that the proposed model has an acceptable rate in DTI prediction and is compatible with the proposed methods in other papers.

## Introduction

Predicting the interactions between drugs and targets is vital in the drug discovery task. Recently, the focus of researchers has been on innovative drug development strategies on the basis of knowledge regarding the available drugs^1^. In order to attain their functions, drugs are generally coated with at least one protein. Therefore, finding out new interactions among drugs and target proteins is pivotal for new drug development, because the misconceived statement of proteins may give rise to drug side effects^2^. Identifying DTIs is highly crucial in discovering and developing new drugs. Due to the high cost and the time required to recognize DTIs experimentally, computational approaches have been suggested which can recognize potential DTIs in order to accelerate developing new drugs^3^. Valuable insights into the function of the drug mechanism are the results of computational approaches for DTI prediction^4^. Computational approaches fall into three categories: Ligand-based approaches, Docking-based approaches and Chemogenomic-based approaches^5^. Each approach has its advantages and disadvantages. Ligand-based approaches are beneficial even in the absence of an empirical 3-dimensional structure. These approaches have high computational complexity and require large amount of data to obtain correct information^6^. Docking-based approaches model the reality more accurately, despite their high computational cost and low scalability. Another advantage of these approaches is that they are as flexible as Ligand-based approaches. These approaches problem is the lack of data 3-dimensional structure. Considering that they require this 3-dimensional structure, Ligand-based approaches are proposed that these approaches will work well even in the case of the lack of data 3-dimensional structure^7^. Third category of computational approaches are chemogenomic-based approaches. One of the advantages of this approaches is that special analogs in drugs can be detected more easily. Another benefit of these approaches is that the coverage of the chemical space is more complete. Moreover, the results obtained from a drug may be used for the discovery of relevant drugs. In addition, using this approach makes attaining structure–activity relationships easier^8^. The basis of the studies on the prediction of DTIs can be one of the methods of machine learning. Machine learning methods in this area include feature based methods (FBM), Kernel based methods (KBM), and Similarity-based methods (SBM)^9^.

Newly, kernel-based methods have been widely applied to identify DTIs. In addition to modeling nonlinear relationships, these methods propose models that can be applied to various data such as stings and time-series data. The problem with these methods is that the proposed models have low interpretability and understanding. Also, if large datasets are applied, these methods are not computationally efficient^10^.

In feature-based approaches, each Drug and protein is represented by a numerical feature vector, which demonstrates the different types of physical, chemical, and molecular features of each of the relevant samples^11^. One of the advantages of feature extraction methods is that they can reveal the intrinsic features of compounds and targets that have a crucial role in DTIs, the outcome of which would be more interpretable^11^.

Feature-based methods are divided into two categories: methods according to deep learning, and classical feature-based methods^12^. The input to deep learning methods is often the protein sequence and the structure of the drug. From this type of data, different features are extracted during different layers. In the end, the prediction of DTIs occurs in the final layer^13,14^.

In^15^ sequence-based deep learning,^16^ deep neural multi-function learning,^17^ deep convolution neural networks,^18^ light deep convolution neural networks,^19^ end-to-end deep learning approaches are applied to predict interactions between drug and target. In using Autoencoders, we can also mention^20^ and^21^ that were done in 2021.

The remaining of the paper is organized as follows. In the next Section, we introduce the related works. Then we explain the method. After that, we report experimental results obtained on different classification. Finally, we draw the conclusions.

## Related works

Numerous computational methods have been developed for DTI prediction problem. In 2021, Jiajie Peng and colleagues used the learning representation graph to provide a framework^22^. In another study, the data needed to predict DTIs were described^1^.

Kernel-based methods are one of the machine learning methods that many people have studied in this field. Muhammad Ammad-ud-din et al. analyzed integrated and personalized QSAR approaches in cancer by kernelized Bayesian Matrix Factorization^23^. In a study conducted in 2018, Anna Cichonska et al. proposed a method with multiple pairwise kernels for effective memory and time learning^24^. Another important category is similarity-based methods^25^. Similarity-based approaches rely on the hypothesis that compounds which are biologically, topologically, and chemically similar, have similar functions and bioactivity, therefore have similar targets^26^. In^27^ a similarity-based monitoring technique was presented to identify the interactions among new drugs and known targets.

In order to predict DTI, a similarity model is proposed, in 2021 that uses two-dimensional CNN in the external products between column vectors corresponding to two similarity matrices in drugs and targets^28^.

There are also various machine learning methods for this prediction. Using multi-tag learning, Seo May et al. represented a framework for predicting interactions^29^. In another work by Nin Metai et al. in 2020, similarity-based methods, as well as machine learning approaches, were used^30^. Although machine learning-based methods have been proven to be effective in identifying DTIs, there are still many challenges:

- Most methods that are in the form of supervised learning have difficulty selecting negative samples.
- Predictive models on the basis of machine learning are usually constructed and evaluated with overly simple experimental settings.
- Most machine learning-based methods have poor descriptive features. Therefore, it is difficult to distinguish a potential drug mechanism from its function considering a pharmacological perspective^31,32^.

More generally, the key challenges in predicting DTIs include the extraction of all critical drug–target features, the issue of data inconsistencies, and data class imbalances during the prediction process. Feature-based methods are one of the machine learning methods that many people have studied in this field. Articles that have been written so far based on feature-based methods for identifying DTIs have often been innovative in four areas: feature extraction, feature selection, balancing and new classifier^33^.

In the field of feature extraction, Cheng Wong et al. tested features with fingerprint for electro topological status of drugs and APAAC of target proteins in 2020^32^. In 2021, a FastUS algorithm was proposed to work with unbalanced data^34^.

In^2^, the features of drugs and proteins are combined to provide the features of per drug-protein pair. In^35^ they has proposed a new predictive method that used the SMOTE method to work with data that is not balanced. In^36^, Zheng Yang et al. applied a new computational model along with the PSHOG gradient and the PSSM matrix for feature extraction. In a 2020 study, a new computational approach was proposed which used the GIST feature^37^. In another study by Zheng Wong et al. in 2020, a useful computational methodology was proposed which applied protein sequence information^38^.

In another study^39^, an efficient computational method was proposed using the Rotation Forest classifier and the LBP feature extraction method in predicting PPIs from the PSSM matrix. In 2019, Hassan Mahmoud et al. proposed a new computational model to identify DTIs^40^. In the realm of proposing a new classifier, Dmitry Karasov et al. proposed an approach providing the Fuzzy classification of target sequences^41^. In another study in 2020, a new DTI prediction method was proposed in which bi-clustering trees were built on reconstructed networks^42^.

In the present methods, no attention has been thrown to the extraction of effective features. While this matter causes a high discrimination quality, an increase in the verification rate, and therefore a higher detection quality. Furthermore, in extracting features, the dimension of the features is high, so this issue is needed to be managed.

Data imbalance is another problem that currently exists. So that unknown interactions are many times more than True-Positive interactions. As a result, the imbalance between the two classes is a challenge that needs to be worked on.

In addition to the challenges that are commonly associated with deep learning-based DTI models, due to the fact that deep learning methods require a large amount of data for network training and also have a high computational load, we have omitted this method in this study. Hence, classical methods have been considered, in which the feature is extracted from the sequence of drug and protein^1,43^.

## Method

**Figure 1:**
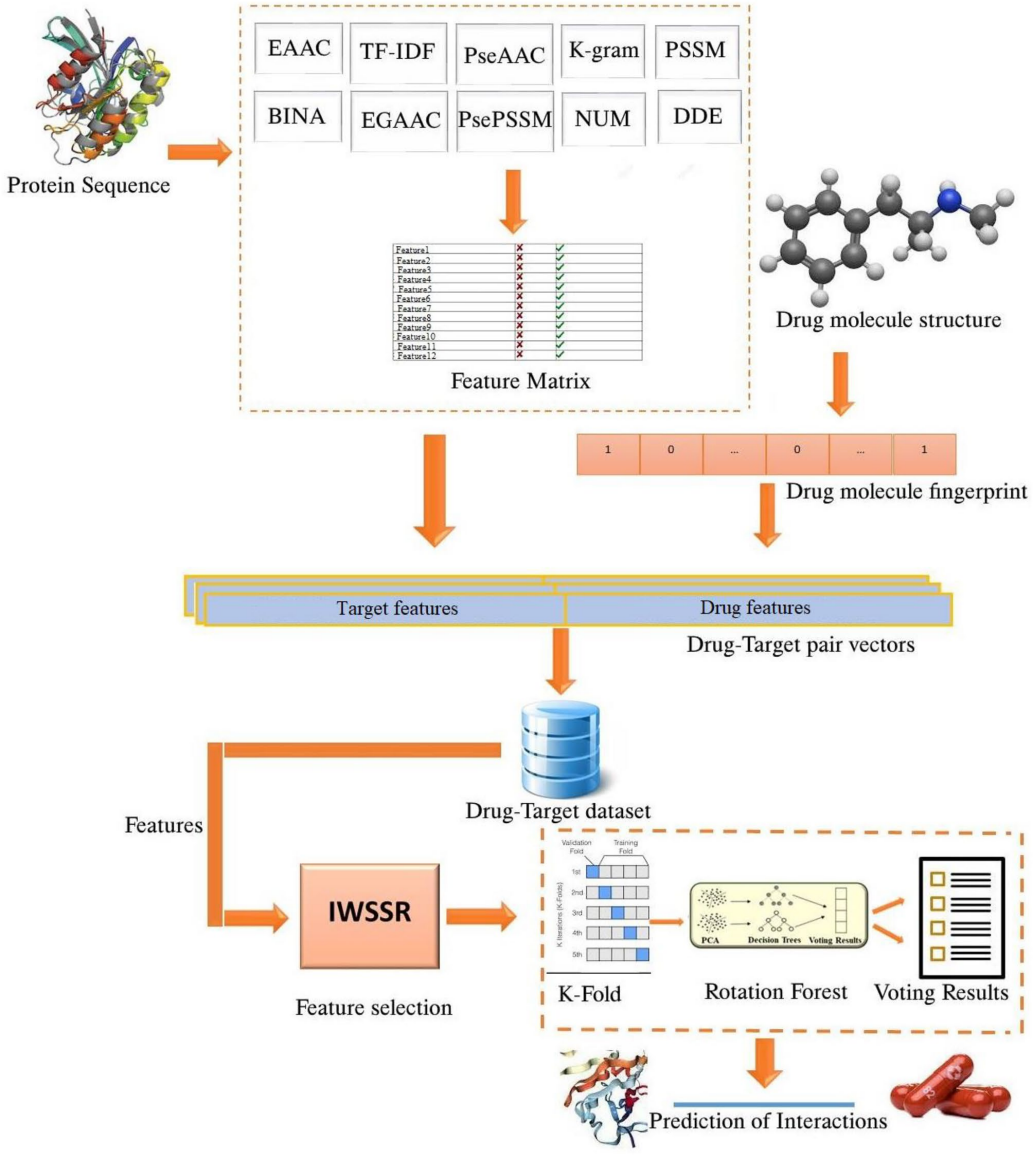
General steps of the proposed method.

In this work, a machine-based learning method is proposed to identify DTIs. In this method, first, different features are extracted from the sequence of proteins, and the feature vector of proteins is formed. Then, a fingerprint is extracted from the structure of the drug. These features are combined, that Due to the high dimension of the features, the features are then selected based on the IWSSR method. Finally, the rotation forest model is then trained to identify interactions. Figure 1 shows the proposed method flowchart. The details of each step are given below.

### Feature extraction

In this step, the information of each sequence is returned to a numeric vector by the use of a feature extraction algorithm. This step is one of the most important steps in classification phase that will directly affect the results of the model prediction. Regarding the fact that this study has two inputs of drug and protein, feature extraction is divided into two categories: feature extraction from drugs and feature extraction from proteins.

### Feature extraction of drugs

Researchers have shown that molecular fingerprints can describe the structure of a drug. The fingerprint of structural relationships shows drugs as the vectors of Boolean substructure through separating the molecular structure of drugs into various sections.

Even though each molecule is divided into separate parts, it preserves the structural information of the entire drug. These descriptors curtail the possibility of information failure and imprudent encounters in the description and screening procedure. In particular, a predefined dictionary that includes all the infrastructures corresponding to the fragments of the drug molecule. In case a fragment is present in the dictionary, its location on the user's device is set to "one"; Otherwise it is considered as "zero". The database of the complete fingerprint creates an effective way for the description of the drug molecular formation in the shape of binary fingerprint vectors. In this paper, a map of the chemical formation derived from the PubChem system at https://pubchem.ncbi.nlm.nih.gov/ is used. This scheme contains 881 molecular infrastructures. Therefore, the descriptors of the structure of drug molecular of features have used the 881-dimensional binary vector format^28^.

### Feature extraction of proteins

One of the most significant phases in identifying DTIs is the extraction of important features from protein sequences. For this purpose, in this paper, various features from protein sequences have been extracted. These features include EAAC, EGAAC, DDE, TF-IDF, k-gram, BINA, PSSM, NUM, PsePSSM, PseAAC. The description and the feature extraction method of each is presented below:

- Enhanced amino acid composition (EAAC)

This method was proposed by Chen et al. In this algorithm, protein sequence information is extracted and the amino acid frequency information is calculated based on it. This method is calculated based on the following equation:

1 $$g\left( {m,n} \right) = \frac{{H\left( {m,n} \right)}}{H\left( n \right)} , m \in \left\{ {1, 2, \ldots , 21} \right\} , n \in \left\{ {W_{1} ,W_{2} , \cdots ,W_{L} } \right\}$$

In this relation, m shows the amino acids, n indicates various windows with different size, H(m,n) is the number of amino acids of type m and H(n) is the window longitude n^44^.

- Enhanced grouped amino acid composition (EGAAC)

In this method, protein sequences are converted to numerical vectors based on their features. This method is an influential feature elicitation algorithm that is applied in bioinformatics study area namely, prediction of malonation sites, etc. 20 different sorts of amino acids are set into five groups regarding five physical and chemical features (physicochemical): The aliphatic group includes GAVLMI amino acids, the aromatic group includes GFYW amino acids, the positively charged group includes KRH amino acids, the negatively charged group includes DE amino acids, and the uncharged group includes STCPNQ amino acids. Depending on the basis of this grouping, the following equation is recommended for the calculation of EGAAC:

2 $$G\left( {g,n} \right) = \frac{{H\left( {g,n} \right)}}{H\left( n \right)} , g \in \left\{ {g_{1} ,g_{2} ,g_{3} ,g_{4} ,g_{5} } \right\} , n \in \left\{ {W_{1} ,W_{2} , \ldots ,W_{L} } \right\}$$

In this formula, H(g,n) demonstrates the number of amino acids in group g in window n and H(n) is equal to window longitude n. In this study, the window size is considered to be L-5 (L is length of proteins sequence)^44^.

- Dipeptide deviation from the expected mean (DDE)

In^45^, which has been studied in the field of feature extraction based on amino acid composition, the Dipeptide Deviation method from the expected mean (DDE) has been proposed and developed in order to distinguish epitopes of a cell from non-epitopes by the use of this feature extraction method. For this purpose, the Dipeptide composition of a protein (DC) sequence is first calculated as follows:

3 $$DC\left( {m,n} \right) = \frac{{H_{mm} }}{H - 1} \;\;\;m,n \in \left\{ {A,C,D, \ldots ,Y} \right\}$$

In this regard, ${H}_{mm}$ is the amino acid pairs number mn and H is the amount of the protein sequence. The second step is to compute the theoretical mean (TM) and theoretical variance (TV) of a protein sequence as follows:

4 $$TM\left( {m,n} \right) = \frac{{C_{m} }}{{C_{H} }} \times \frac{{C_{n} }}{{C_{H} }}$$

In this regard, ${C}_{m}$ is the codons number that encodes the first amino acid and ${C}_{n}$ is the number of codons that encodes the second amino acid, and ${C}_{H}$ is the aggregate of all probable codons.

5 $$TV\left( {m,n} \right) = \frac{{TM\left( {m,n} \right)\left( {1 - TM\left( {m,n} \right)} \right)}}{H - 1}$$

At last, DDE is calculated according to DC, TM and TV values. The computation of the DDE feature vector is as follows^44^:

6 $$DDE\left( {m,n} \right) = \frac{{DC\left( {m,n} \right)\left( {1 - TM\left( {m,n} \right)} \right)}}{{\sqrt {TV\left( {m,n} \right)} }}$$

- Term frequency-inverse document Frequency (TF-IDF)

The TF-IDF feature extraction method consists of two terms: TF, meaning term frequency, and IDF, which is called inverse document frequency. To obtain the TF-IDF equation, each of these two terms must be calculated separately and the product of the two terms must be multiplied. Each of the two terms is calculated as follows: TF (t, d) is the number of repetitions of the amino acid t over the total number of proteins. There are opinions, how to calculate this value as follows:

7 $$IDF\left( t \right) = log \left( {\frac{\left| D \right|}{{DF\left( t \right)}}} \right)$$

After calculating these two terms, the TF-IDF value is obtained based on the following equation^46^:

8 $$TF - IDF\left( t \right) = TF\left( {t,d} \right) \times IDF\left( t \right)$$

- 1-gram

1-g is the specification of k-grams for which k is arranged to 1. The relative frequencies of all 21 sorts of amino acids (20 standard amino acids and the unreal code O when their length are not equal) are computed in 1-g applying the equation which is presented as follows:

9 $$f\left( r \right) = \frac{{N_{r} }}{N} r = 1, 2, \ldots , 21$$

where ${N}_{r}$ designates the number of amino acid r and N designates the longitude of the section. Consequently, a 21-dimensional vector would be achieved for each section^47^.

- 2-gram

2-g computes the relative frequencies of all probable dipeptides in the sequence. The factors of the feature vector are described as:

10 $$f\left( {r,s} \right) = \frac{{N_{rs} }}{N - 1} r,s = 1, 2, \ldots , 21$$

where ${N}_{rs}$ declares the number of the dipeptide rs, N states the longitude of the section and N-1 shows the total number of dipeptides in the encoded section^47^.

- Numerical representation for amino acids (NUM)

NUM aims to reverse sequences of amino acids into sequences of numerical values as by mapping amino acids in an alphabetical range: the 20 standard amino acids are given as 1, 2, 3, …, 20, and the unreal amino acid O is demonstrated as 21^47^.

- BINA

The binary encoding of amino acids transforms per amino acid in a part to a 21-dimensional orthogonal binary vector. Not the same as NUM defined over, BINA indicates per amino acid as a 21-dimensional binary vector encoded by one ‘1’ and 20 ‘0’ factors. For example, alanine (‘A’) is demonstrated as 100,000,000,000,000,000,000, cysteine (‘C’) is demonstrated as 010000000000000000000, etc., when the dummy amino acid ‘O’ is demonstrated as 000000000000000000001^47^.

- PSSM

PSSM, or position-specific scoring matrix, is a kind of scoring matrix applied in BLAST protein surveys, where a score for an amino acid is assigned separately on the basis of its position in a sequence of several proteins. In general, this method extracts evolution-based features.

11 $$PSSM = \left[ {\begin{array}{*{20}c} {P_{1,1 } } & \cdots & {P_{1,20} } \\ \vdots & \ddots & \vdots \\ {p_{L,1} } & \cdots & {P_{L,20} } \\ \end{array} } \right]$$

In this regard, L shows the size of the protein sequence, 20 shows the 20 amino acids, and Pi, j, the possibility of mutation of the amino acid ith to the amino acid jth in the process of biological development. Therefore, PSSM scores are demonstrated as positive or negative integers. Positive scores show that the presented amino acid replacement takes place at a greater rate than is accidentally expected, but negative scores manifest that replacement takes place not more than what is anticipated. PSSM contains protein sequence positional information and evolutionary information^46^.

- PsePSSM

PSSM which is described above, has two major problems as follows:

- As protein sequence length changes, machine learning algorithms cannot handle them directly.
- PSSM does not apply to the sequence order information.

To overcome these two problems, PSSM is replaced by PsePSSM.

PsePSSM or Pseudo Position-Specific Score Matrix can be calculated using the following formulas:

12 $${\text{PsePSSM}} = \left[ {p_{1} , p_{2} , \ldots , p_{20} , p_{1}^{{\upvarepsilon }} , p_{2}^{{\upvarepsilon }} , \ldots , p_{20}^{{\upvarepsilon }} } \right]^{T}$$

13 $$p_{j}^{{\upvarepsilon }} = \frac{1}{{L - {\upvarepsilon }}}\mathop \sum \limits_{i = 1}^{{L - {\upvarepsilon }}} \left[ {P_{i,j} - P_{{i + {\upvarepsilon },j}} } \right]^{2} ,\;\;({\text{j}} = {1},{ 2}, \ldots ,{2}0;\;\;{\upvarepsilon } < {\text{L}}$$

The ${n}_{th}$ rank correlation factor is shown by ${{p}_{j}}^{\mathrm{n}}$ which can be obtained through computing PSSM scores relating to two consecutive Amino Acid residues respecting j in one protein sequence.

$\upvarepsilon$ is related to the amount of rank correlation factor which is needed to be less than the length of the smallest protein sequence^48^.

- PseAAC

The concept of PseAAC or pseudo amino acid composition is representative of the advanced version of AAC. A sequence protein is demonstrated by P, and L represents Amino Acid residues.

PseAAC formula is calculated as follows:

14 $${\text{P}} = [R_{1} R_{2} R_{3} - - - - R_{L} ]$$

AAC is a 20-dimensional array and each element of this array represents the number of each Amino Acid occurrence in the P sequence by the length L.

15 $${\text{P}} = \left[ {f_{1} f_{2} f_{3} \ldots f_{20} } \right]^{T}$$

AAC has the problem of lacking sequence order data. So, when classifying there would be no chance of using a protein sequence. To overcome this problem, PseAAC is recommended which is a set of 20 + λ discrete factors. The first 20 factors in PseAAC can be equal to conventional AAC. Although factors from 20 + 1 to 20 + λ demonstrate various sequence order correlation factors. The number of λ factors can change and relate to the size of functions of Amino Acids that can be collected. Therefore with AAC, features can be elicited on the features such as mass, which can be different for various Amino Acids and can be calculated in the previous studies^49^. Extracted features from protein sequences are listed in Table 1.

**Table 1:** Extracted features from protein sequences.

Row	Feature	Dimension
1	Enhanced amino acid composition (EAAC)	100
2	Enhanced grouped amino acid composition (EGAAC)	25
3	Dipeptide deviation from the expected mean (DDE)	400
4	Term frequency-inverse document frequency (TF-IDF)	20
5	2-g	400
6	Numerical representation for amino acids (NUM)	L
7	BINA	21*L
8	Position-specific score matrix (PSSM)	20*L
9	Pseudo position-specific score matrix (PsePSSM)	220
10	pseudo amino acid composition (PseAAC)	28

### Combination of features

Regarding the fact that the goal is to identify DTI, the features relevant to drug and protein are combined and each pair is considered as a sample. If there is a connection between them, it is labeled "one". Otherwise, the label “zero” is assigned to them.

### Feature selection

Because of the high number of features in each pair of drugs and proteins, giving rise to problems such as time complexity, as well as the possibility of model preprocessing, it is better to select the related features and remove the unrelated ones by the use of feature-selecting methods. Thus, at this stage, the IWSSR method is used to reduce the number of input variables for developing the prediction model. Hence, duplicated, irrelevant, and noisy features are discarded since they enhance the complexity of the model and make it harder to predict DTI. Moreover, they make the training of the model more difficult, and therefore the results of the predictions will not be reliable.

**Figure 2:**
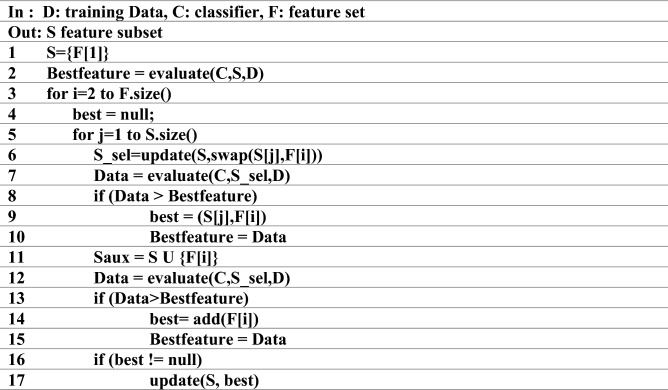
IWSSR pseudo-code algorithm^50^.

In this step, applying the IWSSR hybrid algorithm, the effective features are looked for in the space of features. The IWSSR algorithm, which is an expansion of the IWSS algorithm, is one of the algorithms for selecting a feature subcategory based on the wrapper. In this strategy, first of all, in the filter level, the relationship per feature to the class labels is computed and weight is related to each feature. In IWSSR, the SU standard is applied to weight features. SU is a standard based on nonlinear information theory. This standard assesses each feature separately and allocates a number to each of them in the range of [1 and 0] that indicates the weight of every feature according to its class label. The vast amount shows the great significance of the feature. This standard is computed as follows:

16 $${\text{S}}U_{i,c} (F_{i} ,{\text{C}}) = 2\frac{{H\left( {F_{i} } \right) - H(F_{i} |C)}}{{H\left( {F_{i} } \right) - H\left( C \right)}}$$

where C is the class label, Fi shows the ith feature, and H represents the entropy. Next, in the wrapper step, the features are set in decreasing manner based on their weight. An additional method is then applied to choose a subcategory of features. Figure 2 reveals the pseudo-code of the IWSSR algorithm. In this algorithm, S is the candidate subcategory of the chosen features. Initially, the selected subcategory is empty, and in the first repetition, the feature with the highest rank is joined to the selected subcategory.

After that, a classifier is taught on the basis of the selected subcategory and the training data. Classification accuracy is kept as the greatest outcome obtained. The next step is done in two levels; in the first level, a high-ranking feature that has not been assessed yet is substituted with every feature in the selected set. After per replacement, a new classifier is trained applying the gained subcategory. The accuracy of the classifier is then computed. If the supplement of a recent feature increases the accuracy of the classifier in comparison with the former subcategory, the obtained outcome is retained as the greatest one. In this way, the dependency of the selected feature is measured with the previously chosen features, and if it is not dependent on any of the chosen features, it will be joined to the selected subcategory. In the next level, the investigated feature (the feature that was substituted by the features of the chosen subcategory in the first level) is joint to the chosen subcategory S (gained in the preceding level) and a recent classifier is trained on the basis of the recent subcategory, and the accuracy of the classifier is computed. If the accuracy of the subcategory is better than the accuracy of the elected subcategory in the first level, it will be kept as the greatest obtained outcome. After the first and second levels, if we achieve a greater subcategory in every level, the most satisfactory subcategory is chosen as the subcategory of this cycle (repetition) and the desired feature is used in the chosen subcategory^50^.

### Classification of features

**Figure 3:**
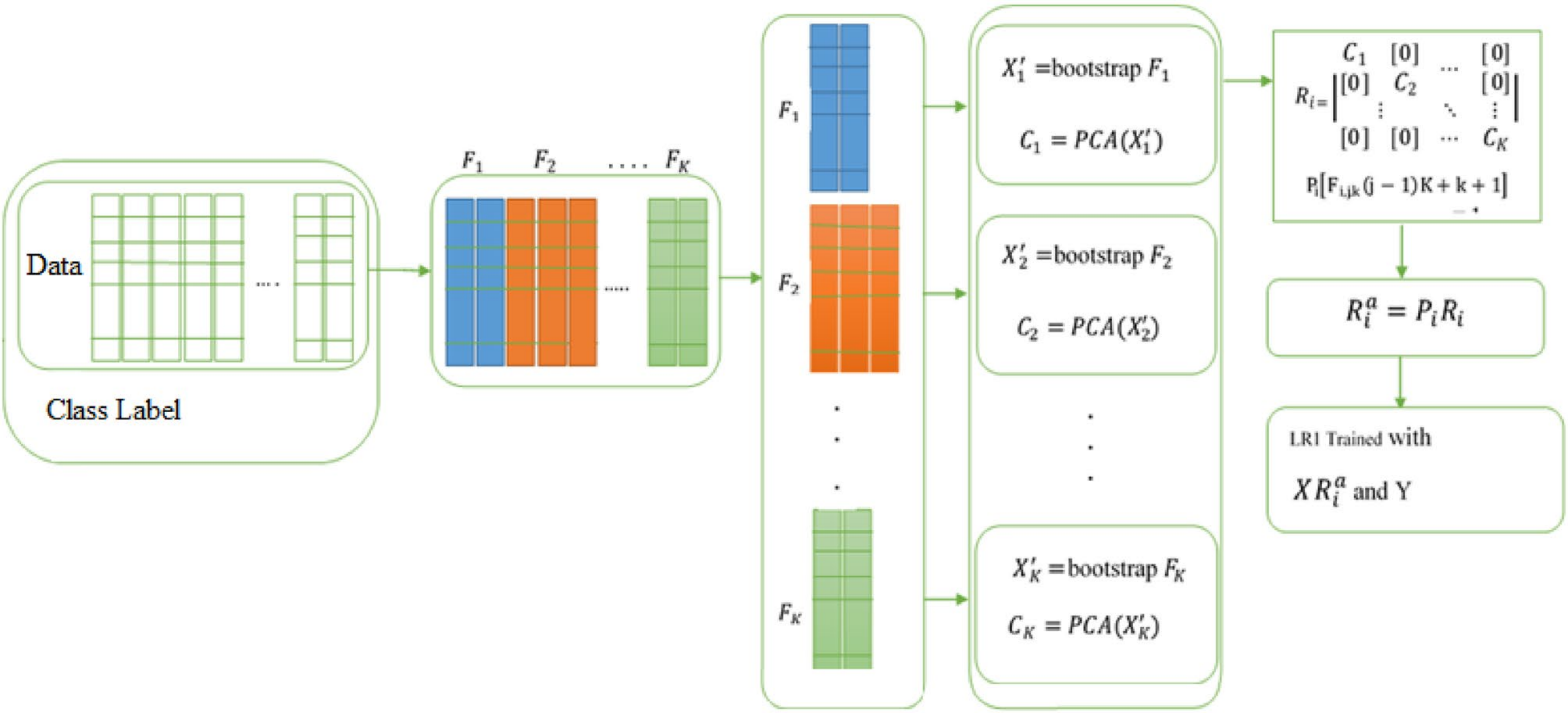
Rotation forest^51^.

The classifier used in this article is Rotation Forest. Due to the fact that this classifier has diverse parameters to be adjusted, the Cross-validation K-Fold method or passing evaluation is used to adjust the parameters of the classification model. Rotation Forest is a classification method that is mainly applied in supervised learning. This method was first offered by Rodriguez et al.^35^ and its prophesy accuracy is similar to that of an Ensemble learning classifier. In the Rotation Forest algorithm, the feature set S is split into K size of subcategories by chance, and the Bootstrap prototyping technique is used to train 75% of the genuine samples in every feature subcategory so that the sparse rotation matrix is obtained. The classifier is then built in several steps applying matrix features. The work of the Rotation Forest algorithm is on the basis of feature transfer and feature selection, and concentrates on improving the accuracy and the difference of the base classifiers. The Principal Component Analysis (PCA) method is applied to do feature deformation in all the split subcategories whose aim is to store data effectively. Not only does this method distinguish per subcategory from the other, but it also plays an important task in data preprocessing. Thus, Rotation Forest can develop Ensemble variety and increase the accuracy of the foundation classifier. Assume that W = [${W}_{1}$, ${W}_{2}$,…, ${W}_{n}$] includes n features of a sample. We consider W as a set of training samples whose amount is N * n. N indicates the number of samples. Assume H as a range of features, assuming the corresponding label is Y = [${Y}_{1}$, ${Y}_{2}$,…, ${Y}_{n}$] ^ T. The feature set is split into K non uniform subcategories by chance. Assume that the number of decision trees is equal to L, which can be represented as ${T}_{1}$, ${T}_{2}$,…, ${T}_{L}$, respectively. The steps for building a Rotation Forest classifier are as follows (Fig. 3):

1. Choose the appropriate parameter for K; the feature set H is split into K subcategory (s) by chance where per subcategory includes (n/K) features.
2. $${H}_{ij}$$ represents the $${j}_{th}$$ subcategory of the training subcategory that is applied to train the ith classifier ($${T}_{i})$$. For every subcategory, a recent $${W}_{ij}$$ training set is made after a re-sampling from bootstrap, with 75% of the W training set.
3. To produce the coefficients in the effective ${P}_{ij}$ matrix, principal component analysis (PCA) is used on ${W}_{ij}$ that is an M * 1 matrix. ${P}_{ij}$ is displayed as ${B}_{ij}$ (1),…, ${B}_{ij}$ (${M}_{j}$).
4. The coefficients obtained in the ${P}_{ij}$ matrix have formed a sparse rotation matrix called ${R}_{i}$, which is shown below:
  17 $$Ri = \left[ {\begin{array}{*{20}l} {b_{i1}^{(1)} , \ldots b_{i1}^{{(M_{1} )}} } \hfill & 0 \hfill & \cdots \hfill & 0 \hfill \\ 0 \hfill & {b_{i2}^{(1)} , \ldots b_{i2}^{{(M_{2} )}} } \hfill & \cdots \hfill & 0 \hfill \\ \vdots \hfill & \vdots \hfill & \ddots \hfill & \vdots \hfill \\ 0 \hfill & 0 \hfill & \cdots \hfill & {b_{iK}^{(1)} , \ldots b_{iK}^{{(M_{K} )}} } \hfill \\ \end{array} } \right]$$

At the time of prediction, using the sample ω, ${d}_{ij}$ in (x ${R}_{i}^{a}$) is considered as a probability that predicts whether ω belongs to λj or not by using the Ti classifier. Then the level of trust in the class is calculated using the average combination, the formula of which is as follows:

18 $$\lambda_{j} (\omega ) = \frac{1}{L}\mathop \sum \limits_{i = 1}^{L} d_{ij} { }({\text{x}}R_{i}^{a} )$$

The category with the highest probability will be considered as a test sample x^36,37^.

### Predicting the new DTI

The final step is to predict interactions. In the end, after training the Rotation Forest model, the model is used to predict the new DTI. On the basis of the chosen evaluation criteria, which are described in detail in “The Results” section, acceptable results have been obtained from this step.

## The results

### Evaluation criteria

In this paper, we have applied 4 evaluation criteria to evaluate the efficiency of the proposed method. These criteria include accuracy (Acc), sensitivity (Sen), precision (Pre), and Matthew correlation coefficient (MCC), which are calculated as follows:

19 $${\text{Acc}} = \frac{TN + TP}{{TN + FN + TP + FP}}$$

20 $${\text{Sen}} = \frac{TP}{{FN + TP}}$$

21 $${\text{Pre}} = \frac{TP}{{FP + TP}}$$

22 $${\text{MCC}} = \frac{TN*TP - FN*FP}{{\sqrt {\left( {TN + FN} \right)*\left( {TP + FP} \right)*\left( {TN + FP} \right)*\left( {TP + FN} \right)} }}$$

In addition, Receiver Operating Characteristic curves (ROCs) have been used to describe the results, and the space under the curve (AUC) has been computed to confirm the possibility of making predictions^36^.

### Data set

This study has applied the Gold Standard data set utilized by Yamanishi et al.^52^ as a Benchmark dataset downloaded from http://web.kuicr.kyoto-u.ac.jp/supp/yoshi/drugtarget/. In the Gold Standard Database, information on DTIs is gained from the KEGG BRITE, BRENDA, Super-Target, and DrugBank datasets. This dataset is split into four major datasets including enzymes, ion channels (IC), G-protein-coupled receptors (GPCR), and nuclear receptors (NR). The number of understood drugs in these datasets are 445, 210, 223, and 54, in the order given; and the number of known proteins in these datasets are 664, 204, 95, and 26, in the order given. After precise testing of these drugs and proteins, an amount of 5,127 pairs of DTIs were gained, and the number of interactions between drug and protein couples known so far in each dataset was 2926, 1476, 635, and 90, in the order given. Extended information on drugs and proteins is available from the KEGG database before further analysis^53,54^. Each protein is displayed using an amino acid sequence and after that stored in a text file. The chemical form of every drug molecule is converted to the Mol file format, after which the file format is downloaded. The information of the datasets applied in this article is presented in Table 2^35^.

**Table 2:** Database information used in this article^35^.

Dataset	Drug	Protein	Interaction
Enzyme	445	664	2926
IC	210	204	1476
GPCR	223	95	635
NR	54	26	90

### Results from different features

As stated above, in order to predict DTIs precisely, different features must be extracted from the protein-drug sequence. Given that the purpose of this paper is to extract the effective features of the protein sequence, the extracted features are analyzed in this section. In this paper, 10 feature-extraction methods are applied to protein sequences and extract different kinds of protein features.

In order to evaluate the extracted features by each method, the rotation forest model is trained using each of the EAAC, EGAAC, DDE, TF-IDF, K-gram, BINA, PSSM, PsePSSM, PseAAC and NUM features on the basis of cross-validation with the value of k = 10. The results of this experiment are demonstrated on Enzyme data set in Table 3.

**Table 3:** System efficiency criteria for different features.

	EGAAC	EAAC	DDE	TF-IDF	K-gram	BINA	PSSM	NUM	PsePSSM	PseAAC
	${f}^{1}$	${f}^{2}$	${f}^{3}$	${f}^{4}$	${f}^{5}$	${f}^{6}$	${f}^{7}$	${f}^{8}$	${f}^{9}$	${f}^{10}$
Accuracy	84.46	81.86	76.47	80.74	80.83	86.74	87.23	86.45	**88.18**	84.43
Sensitivity	79.13	77.2	71.91	76.71	76.84	81.04	83.54	82.23	**84.23**	82.31
Specificity	88.45	85.35	79.88	83.77	83.82	**89.87**	88.27	88.67	89.09	86.71
Balance rate	83.79	81.28	75.9	80.24	80.33	84.79	85.9	85.45	**86.68**	84.23

Significant values are in bold.

As evident in Table 3, the features extracted by PsePSSM have greater differentiating power and have a higher detection rate in the whole data set. Moreover, PSSM, PseAAC and BINA methods have acceptable performance too. Each of these features represents a pattern of data that makes the classification model identify interactions well.

**Figure 4:**
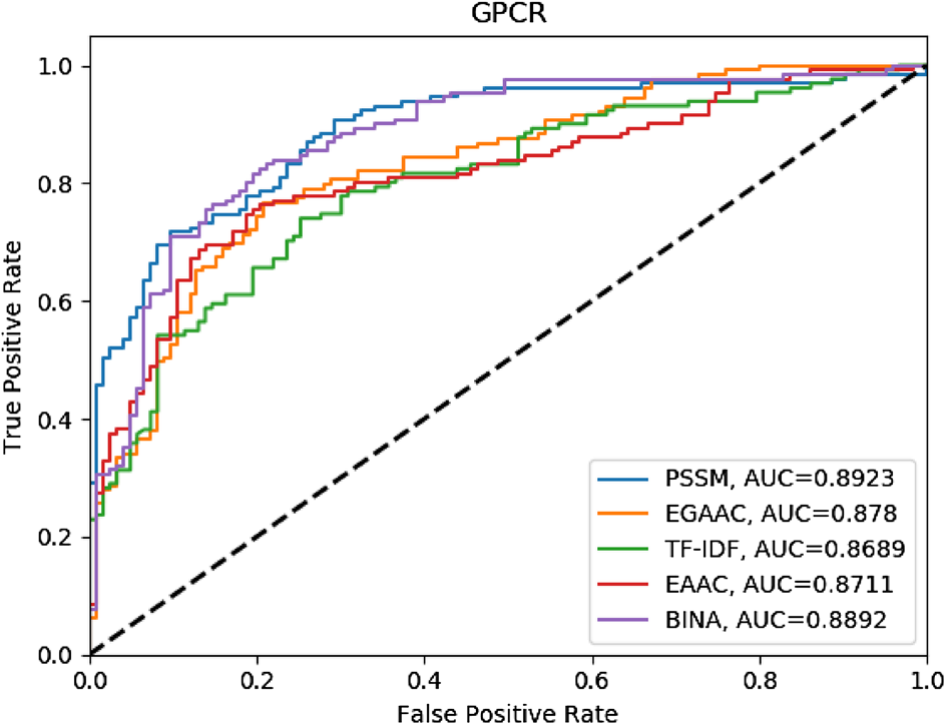
ROC diagram for the comparison of the five features.

In order to compare the extracted features, the ROC diagram in Fig. 4 is drawn for 5 types of features by the use of different methods. In this diagram, it is also obvious that the PSSM feature performs better than the other ones and has a higher area under the diagram. The TF-IDF method had lower performance compared with the other methods. On the basis of the results of Fig. 4 and Table 3, it can be concluded that the combination of diverse features improves the performance of the classification model in identifying DTIs.

For this purpose, the extracted features are combined in various modes, and the classifier is trained and tested on the basis of the combination of features. Among the various modes, three had better performance. In the first mode, the features related to the methods (PSSM, EGAAC, EAAC) are combined and the resulting feature vector has 2125 features. In the second mode, the features relevant to PSSM, EGAAC, EAAC, DDE, BINA methods are combined and the feature vector length is 4625, and in the third mode, the features pertinent to PSSM, EGAAC, EAAC, DDE, BINA, K-gram, TF-IDF, NUM, PsePSSM, PseAAC methods are combined. In this mode, the resulting feature vector length contains 6293 features. As it is evident, in all these three modes, the performance of the classification model is greater than the mode before the combination of features. This indicates that the variety of features increases the efficiency of the models. On the other side, in the second mode, the performance of most classification methods is better than that of the third ones. In the second mode, the features are combined well. However, in the first one, there are still some related features that are not included in the combination; hence, the accuracy of the model does not increase much. In addition, in the third mode, since the number of features shows an excessive increase, the model has been over-fitted and the accuracy of the model has been decreased. Therefore, it is better to identify the effective and relevant features and remove the unrelated and noise ones via selecting features. Table 4 shows the results on different categories, without feature selection. The comparison has done on SVM^32^, RF^35^, XGBoost^55^, and DNN^13^ classifiers.

**Table 4:** Comparison of efficiency criteria of various classifications of different features, without feature selection.

Dataset	Combination	Classifier	Acc(%)	Sp (%)	Sn (%)	MCC	AUC
Enzyme	PSSM,EGAAC, EAAC	SVM	59.33	57.75	60.91	0.6839	**0.7223**
		XGBoost	91.44	93.78	93.45	0.8902	**0.9695**
		RF	**95.18**	**95.21**	**93.03**	**0.9049**	0.981
		DNN	68.63	51.14	86.09	0.7348	**0.7891**
	PSSM,EGAAC, EAAC, DDE,BINA	SVM	67.03	65.73	68.34	0.8261	**0.8834**
		XGBoost	96.69	95.79	93.59	0.9151	**0.9711**
		RF	**97.21**	**95.72**	**94.31**	**0.9279**	0.9768
		DNN	92.99	93.52	92.46	0.9023	**0.9649**
	PSSM,EGAAC, EAAC, DDE,NUM, K-gram,TF-IDF, BINA	SVM	66.83	64.84	68.83	0.8311	**0.8714**
		XGBoost	96.69	95.38	93.27	0.9147	**0.9723**
		RF	**97.22**	**95.78**	**94.64**	**0.9311**	0.9781
		DNN	90.10	80.92	99.25	0.8973	**0.9578**
Ion channel	PSSM,EGAAC, EAAC	SVM	65.90	65.38	66.42	0.7482	**0.8831**
		XGBoost	92.15	93.42	90.88	0.9087	**0.9489**
		RF	**94.18**	**95.08**	**96.18**	**0.9234**	0.9634
		DNN	77.99	68.22	87.75	0.7841	**0.9043**
	PSSM,EGAAC, EAAC, DDE,BINA	SVM	69.86	71.04	68.67	0.8418	**0.8931**
		XGBoost	93.24	94.02	91.76	0.9287	**0.9528**
		RF	**95.22**	**97.14**	**97.32**	**0.9448**	0.9749
		DNN	91.17	94.89	94.36	0.9142	**0.9328**
	PSSM,EGAAC, EAAC, DDE,NUM, K-gram,TF-IDF, BINA	SVM	68.66	69.45	67.87	0.8346	**0.8911**
		XGBoost	92.73	93.61	91.24	0.8971	**0.9518**
		RF	**94.71**	**96.42**	**97.10**	**0.9371**	0.9659
		DNN	91.45	92.61	93.45	0.9017	**0.9503**
GPCR	PSSM,EGAAC, EAAC	SVM	64.37	63.58	65.16	0.7934	**0.8942**
		XGBoost	91.46	93.02	91.90	0.8872	**0.9537**
		RF	**92.88**	**93.78**	**95.23**	**0.8943**	0.9644
		DNN	72.85	56.56	89.14	0.8136	**0.8993**
	PSSM,EGAAC, EAAC, DDE,BINA	SVM	71.74	72.56	70.91	0.8623	**0.9061**
		XGBoost	92.76	94.48	93.23	0.9023	**0.9573**
		RF	**94.31**	**95.34**	**96.47**	**0.9217**	0.9721
		DNN	90.87	94.09	92.43	0.8983	**0.9382**
	PSSM,EGAAC, EAAC, DDE,NUM, K-gram,TF-IDF, BINA	SVM	70.95	71.41	70.49	0.8582	**0.9035**
		XGBoost	92.21	93.47	93.82	0.8991	**0.9548**
		RF	**93.29**	**94.62**	**95.93**	**0.915**	0.9692
		DNN	91.15	94.56	93.72	0.8932	**0.9376**
Nuclear receptors	PSSM,EGAAC, EAAC	SVM	70.95	71.41	70.49	0.8582	**0.9035**
		XGBoost	92.21	93.47	93.82	0.8991	**0.9548**
		RF	**93.29**	**94.62**	**95.93**	**0.915**	0.9692
		DNN	91.15	94.56	93.72	0.8932	**0.9376**
	PSSM,EGAAC, EAAC, DDE,BINA	SVM	70.95	71.41	70.49	0.8582	**0.9035**
		XGBoost	92.21	93.47	93.82	0.8991	**0.9548**
		RF	**93.29**	**94.62**	**95.93**	**0.915**	0.9692
		DNN	91.15	94.56	93.72	0.8932	**0.9376**
	PSSM,EGAAC, EAAC, DDE,NUM, K-gram,TF-IDF, BINA	SVM	70.95	71.41	70.49	0.8582	**0.9035**
		XGBoost	92.21	93.47	93.82	0.8991	**0.9548**
		RF	**93.29**	**94.62**	**95.93**	**0.915**	0.9692
		DNN	91.15	94.56	93.72	0.8932	**0.9376**

Significant values are in bold.

As evident in Table 4, all features are combined with the purpose of selecting the effective ones. Then, important features are selected using the IWSSR method. The number of the selected features varies in different datasets. By the use of the IWSSR method, 22 features have been selected in the enzyme dataset, 30 features in the ion channel dataset, 27 features in the GPCR dataset, and 18 features in the nuclear receptor set. This number of features is much less compared with the main ones. In addition, the performance of the classification model is substantially enhanced on various datasets. This indicates that the IWSSR method has prevented the over-fitting of the classification models and has selected the related features in the prediction of interactions. Table 5 shows the results of feature selection on different classifiers.

**Table 5:** Comparison of efficiency criteria of various classifications of different features, with feature selection.

Dataset	Combination	Classifier	Acc (%)	Sp (%)	Sn (%)	MCC	AUC
Enzyme	PSSM,EGAAC, EAAC	SVM	68.18	66.23	67.23	0.6839	**0.7223**
		XGBoost	94.37	95.11	95.22	0.9231	**0.9741**
		RF	**96.41**	**97.11**	**96.76**	**0.9523**	0.9871
		DNN	73.42	62.17	86.21	0.7672	**0.7934**
	PSSM,EGAAC, EAAC, DDE,BINA	SVM	71.12	69.18	72.5	0.8532	**0.9213**
		XGBoost	97.12	96.4	94.89	0.9437	**0.9817**
		RF	**97.67**	**96.21**	**96.64**	**0.9582**	0.9835
		DNN	94.89	95.71	94.82	0.9348	**0.9782**
	PSSM,EGAAC, EAAC, DDE,NUM, K-gram,TF-IDF, BINA	SVM	73.42	76.08	72.45	0.8632	**0.8941**
		XGBoost	98.1	97.26	95.16	0.9461	**0.9847**
		RF	**98.12**	**98.74**	**98.02**	**0.9921**	0.9982
		DNN	92.09	88.46	97.25	0.9217	**0.9709**
Ion channel	PSSM,EGAAC, EAAC	SVM	68.72	69.18	70.06	0.7731	**0.8977**
		**XGBoost**	**94.22**	**95.82**	**93.4**	**0.9307**	**0.9632**
		**RF**	**96.18**	**97.28**	**97.73**	**0.9486**	**0.9736**
		DNN	81.12	72.41	88.43	0.8022	**0.9215**
	PSSM,EGAAC, EAAC, DDE,BINA	SVM	71.97	74.37	72.86	0.8899	**0.9128**
		XGBoost	95.47	96.72	94.38	0.948	**0.9735**
		**RF**	**96.89**	**97.74**	**98.07**	**0.9511**	**0.9807**
		DNN	93.47	95.82	96.09	0.9348	**0.9572**
	PSSM,EGAAC, EAAC, DDE,NUM, K-gram,TF-IDF, BINA	SVM	72.81	73.47	72.81	0.8523	**0.926**
		XGBoost	95.71	96.89	95.12	0.9541	**0.9773**
		RF	**98.07**	**98.6**	**98.42**	**95.42**	0.9911
		DNN	93.86	96.11	96.44	0.9385	**0.9617**
GPCR	PSSM,EGAAC, EAAC	SVM	66.37	64.7	68.34	0.8237	**0.8872**
		XGBoost	93.72	94.29	92.63	0.9145	**0.9608**
		**RF**	**93.78**	**95.56**	**96.48**	**0.9138**	**0.9742**
		DNN	75.23	77.81	89.11	0.8173	**0.9217**
	PSSM,EGAAC, EAAC, DDE,BINA	SVM	73.82	75.18	73.69	0.8943	**0.9243**
		XGBoost	93.71	95.82	94.63	0.9137	**0.9682**
		**RF**	**95.38**	**96.73**	**97.28**	**0.9381**	**0.9792**
		DNN	92.39	95.6	93.52	0.9187	**0.9558**
	PSSM,EGAAC, EAAC, DDE,NUM, K-gram,TF-IDF, BINA	SVM	75.12	74.52	73.94	0.8853	**0.9275**
		XGBoost	94.58	94.89	95.23	0.9228	**0.9783**
		RF	**96.82**	**98.17**	**97.33**	**94.32**	0.9925
		DNN	93.36	96.58	95.68	0.8272	**0.9632**
Nuclear receptors	PSSM,EGAAC, EAAC	SVM	72.65	73.68	72.65	0.8817	**0.9172**
		XGBoost	93.41	94.18	91.88	0.9167	**0.9637**
		**RF**	**93.76**	**95.47**	**93.35**	**0.9273**	**0.9611**
		DNN	91.43	95.83	93.59	0.9156	**0.9477**
	PSSM, EGAAC, EAAC, DDE, BINA	SVM	73.48	74.51	73.36	0.8943	**0.9135**
		XGBoost	94.37	95.89	92.83	0.9248	**0.9634**
		**RF**	**94.8**	**95.71**	**93.27**	**0.9241**	**0.9645**
		DNN	92.73	95.59	93.88	0.9208	**0.9486**
	PSSM,EGAAC, EAAC, DDE,NUM, K-gram,TF-IDF, BINA	SVM	74.69	75.92	74.46	0.9028	**0.9145**
		XGBoost	94.78	94.92	93.08	0.9351	**0.9639**
		RF	**95.64**	**96.75**	**94.78**	**93.08**	0.9653
		DNN	93.39	95.21	94.09	0.9274	**0.9403**

Significant values are in bold.

**Figure 5:**
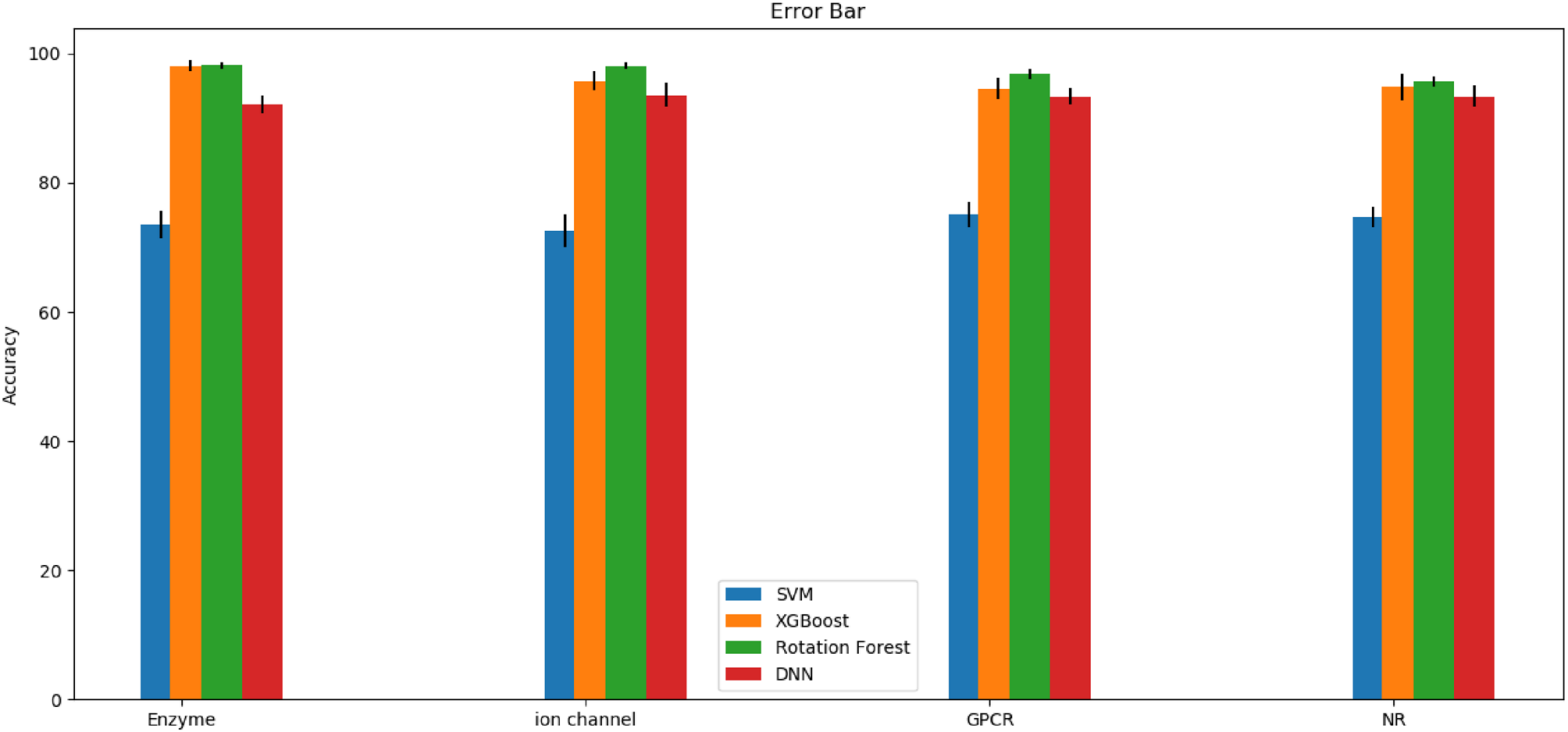
Studying classification models based on error bars for underlying datasets.

Error analysis is carried out to show stability and resistivity of the model. The error bar shows estimated errors in order to attain a deeper understanding of the measurements. Generally, error bars are utilized to show the standard error, standard deviation, or minimum/maximum values in a data. The size of the error bars shows the uncertainty in the measurements. A small error bar indicates the certainty and significance of the measurements whilst a long error bar addresses sparsity and a lesser number of data values. The accuracies of the models via a tenfold cross-validation are showed out in Fig. 5 for the underlying datasets. As evident from Fig. 5, RF has outperformed the others, and SVM and DNN depicts the highest error regarding the lengths of the bars. This shows that RF results are more reliable and meaningful.

**Figure 6:**
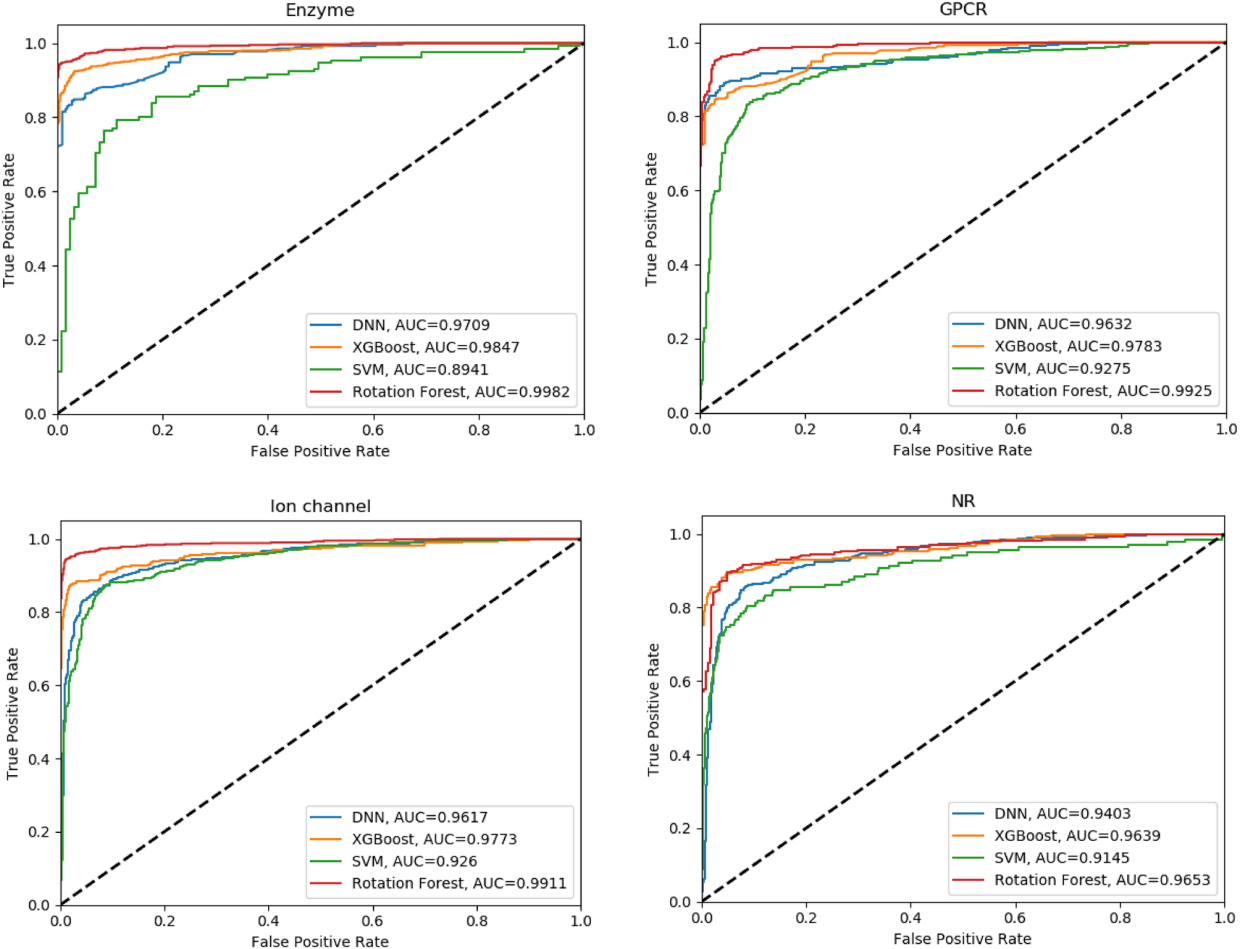
ROC curves of different classifiers on the data sets.

For better evaluation, the proposed method, AUROC curves for different classifiers on the basis of the proposed features are shown in Fig. 6, respectively. As it is clear from the results, on the basis of the selected features, the Rotation Forest classifier has a better performance in comparison with the other methods. This is because the selected features have a good distinguishing feature. In addition, since the Rotation Forest classifier selects the most suitable features for constructing trees, it turns out to be well-generalizable. According to the figures, it is apparent that other classifiers have acceptable performance as well.

In order to better evaluation, in this paper, each dataset is divided into two datasets; a test dataset and an independent dataset. 90% of the original data is chosen randomly for the training and test dataset and 10% for the independent dataset. For this purpose, the training dataset is used to train, and test data is used to evaluate and justify the proposed method, and the independent dataset is applied for final performance evaluation of the proposed method. The results of these experiments are shown in Table 6. The results approve that the proposed method is robust and it has high accuracy rate. Therefore, the method can be used to classify new-drug, new-target, and new drug-new target with high accuracy.

**Table 6:** Performance results of proposed method in test and independent data.

	Test data				Independent data			
	Accuracy	Specificity	Sensitivity	Balance rates	Accuracy	Specificity	Sensitivity	Balance rates
Enzyme	**98.02**	**98.74**	**98.12**	98.24	**97.89**	**98.77**	**98.05**	98.11
ion channel	**98.42**	**98.6**	**98.07**	98.17	**98.23**	**98.53**	**98.1**	98.09
GPCR	**97.33**	**98.17**	**96.82**	97.64	**97.35**	**98.21**	**96.75**	97.61
nuclear receptor	**94.78**	**96.75**	**95.64**	95.66	**94.41**	**96.6**	**95.53**	95.48

Significant values are in bold.

### Comparison with other methods

For better evaluation, the proposed method has been compared to the other available methods that have utilized the mentioned data set. The results of this experiment are shown in Table 7. The compared methods have extracted various features from the protein sequence and used different classifiers. As evident, the values of Acc, Sn, Sp, and MCC of the proposed method are the best ones. In the enzyme dataset, the proposed accuracy rate is 98.12, which is at least 0.8 and at most 9% better than the other methods. This efficiency can also be seen in other data sets. This represents that the extracted and selected features have absolutely good differentiating power.

**Table 7:** Comparison of efficiency criteria of the proposed method and the results reported in the valid articles.

Dataset	Methods	Acc (%)	Sp (%)	Sn (%)	MCC	AUC	AUPR
Enzyme	^4^	89.25	87.48	90.70	80.80	0.9479	**0.8763**
	^3^	88*:*96	90*:*01	87*:*92	77*:*93	0.9509	
	^2^	98.09	98.51	97.66		0.9982	**0.9983**
	^1^	89.15	91.06	86.85	80.65	0.9466	
	Proposed method	98.12	98.74	98.02	90.38	0.9921	**0.9982**
Ion channel	^4^	85.93	86.35	85.38	75.84	0.9312	
	^3^	86*:*37	86*:*24	86*:*45	72*:*72	0.9284	
	^2^	97.32	97.93	96.71		0.9965	**0.9964**
	^1^	86.01	85.66	86.62	75.94	0.9152	
	Proposed method	98.07	98.6	98.42	95.42	0.9911	**0.9974**
GPCR	^4^	82.36	83.35	81.22	70.92	0.8879	**0.8010**
	^3^	82*:*88	83*:*32	82*:*53	65*:*78	0.9040	
	^2^	95.69	96.11	95.26		0.9918	**0.9913**
	^1^	82.20	82.83	81.28	70.62	0.865	
	Proposed method	96.82	98.17	97.33	94.32	0.9925	**0.9921**
Nuclear receptor	^4^	73.89	73.82	75.83	60.15	0.8011	**0.7299**
	^3^	76*:*92	71*:*04	82*:*97	54*:*94	0.8486	
	^2^	94.88	95.81	93.85		0.9559	**0.9867**
	^1^	71.67	69.61	76.45	57.97	0.7795	
	Proposed method	95.64	96.75	94.78	93.08	0.9653	**0.9872**

Significant values are in bold.

One of the reasons that our proposed method is better, compared to other methods, is that our method offers better features by defining and selecting the features that end in more accurate results. In fact, our method observes specificity and sensibility and also considers balance in classes. Hence, bias is not towards the majority class. Unlike Reference 4, where one of its specificity is 87 and its sensibility is 90, in our method, these two do not make so much difference. That is, it doesn’t care what data is used.

## Conclusion

In this paper, a DTI prediction based on protein features, using wrapper feature selection was proposed. This machine learning model consisted of three phases, including feature extraction, feature selection, and classification. In the first phase, it would extract different features such as EAAC, PSSM and etc. from sequence of proteins information and fingerprint information from drugs. These extracted features would then be combined. In the next step, one of the wrapper feature selection methods named IWSSR, due to the large amount of extracted data, is applied. The selected features are then given to Rotation Forest classifier, to have more efficient prediction. Actually, the innovation of our work is that we define the features; and then select a feature selection method such as IWSSR. The results of experiments indicate that the proposed model has an acceptable rate in DTI prediction and is compatible with the proposed methods in other papers.

## Data Availability

This study has applied the Gold Standard data set utilized by Yamanishi et al.^52^ as a Benchmark dataset downloaded from http://web.kuicr.kyoto-u.ac.jp/supp/yoshi/drugtarget/.
